# Virtual reality-based simulation learning on geriatric oral health care for nursing students: a pilot study

**DOI:** 10.1186/s12903-024-04249-y

**Published:** 2024-05-28

**Authors:** Pei-Chao Lin, Shu-Fen Wung, Pei-Chen Lin, Yi-Ching Lin, Cheng-Yu Lin, Hsiao-Ling Huang

**Affiliations:** 1 School of Nursing, College of Nursing, Kaohsiung Medical University, Kaohsiung, Taiwan; 2 Department of Medical Research, Kaohsiung Medical University Hospital, Kaohsiung Medical University, Kaohsiung, Taiwan; 3 Center for Long-Term Care Research, Kaohsiung Medical University, Kaohsiung, Taiwan; 4 College of Nursing, The University of Arizona, Tucson, AZ USA; 5 Department of Oral Hygiene, College of Dental Medicine, Kaohsiung Medical University, Kaohsiung, Taiwan; 6 School of Dentistry, College of Dental Medicine, Kaohsiung Medical University, Kaohsiung, Taiwan; 7 Department of Oral Hygiene, Shu-Zen Junior College of Medicine and Management, Kaohsiung, Taiwan; 8 Department of Radio, TV & Film, Shih Hsin University, Taipei, Taiwan

**Keywords:** Attitude, Intention, Knowledge, Nursing student, Oral health, Self-efficacy, Taiwan, Virtual reality

## Abstract

**Background:**

There is a great need for training and education in the nursing curriculum to improve nurses’ knowledge and skills to provide oral health care.

**Methods:**

A pilot study was conducted to evaluate the use of a virtual reality (VR)-based Oral Health Care Learning System to train geriatric oral health care among nursing students. Fifty undergraduate nursing students were randomly assigned to experimental (*n* = 25) and control (*n* = 25) groups. The experimental group received the VR-based simulation training on geriatric oral health care and the training was implemented twice at two weeks apart from March to November 2021. The control group did not receive the training intervention. Knowledge, attitude, and self-efficacy of geriatric oral health care as well as the intention to assist oral health care for older adults were assessed at the beginning, second, and fourth weeks. Generalized estimating equations were used to analyze the effectiveness of the VR-based simulation training.

**Results:**

After the first round of training, students in the experimental group had significantly greater improvements in knowledge and self-efficacy of geriatric oral health care than in the control group. After the second round of training, students in the experimental group had significantly greater improvements in knowledge, attitude, and self-efficacy of geriatric oral health care as well as the intention to assist oral health care for older adult than in the control group.

**Conclusions:**

The VR-based simulation training was effective to improve undergraduate nursing students’ knowledge, attitudes and self-efficacy of geriatric oral health as well as the intention to assist oral health care for older adults. The VR-based simulation learning system is an effective tool to provide practice experiences to build confidence and skills and to bridge the gap of understudied geriatric oral health content in entry-level nursing curricula.

**Trial Registration:**

ClinicalTrials.gov (NCT05248542; registration date 21/02/2022).

**Supplementary Information:**

The online version contains supplementary material available at 10.1186/s12903-024-04249-y.

## Background

Worldwide, the oral health of the elderly population is poor and the prevalent dental caries and periodontal disease in the elderly have called for public health action [1]. There is also a profound disparity in oral health, even in high-income countries [2]. Poor oral health, such as periodontal disease, has a statistically significant impact on morbidity and mortality as it can lead to malnutrition and frailty [3–5], as well as other systemic health conditions, including cardiovascular diseases, aspiration pneumonia, and diabetes mellitus [6–8]. Poor oral health can also negatively affect older adults’ quality of life due to poor chewing ability and orofacial pain [9].

Good oral health can reduce infections and associated complications and is a critical component of healthy aging [10]. Healthcare providers can play an important role to promote good oral health and preventing oral infections by providing advice and care regarding routine dental health to older adults [11]. Nurses are the largest workforce in healthcare and provide the most direct care. Patient safety initiatives recommend that oral health should be a part of routine patient care and a priority in daily nursing care [12]. Evidence supports that intervention from nurses in oral healthcare leads to improved health outcomes, particularly amongst patients with special healthcare needs. In intensive care units, oral health is identified as a critical nursing activity and the benefits of oral health in maintaining the health and well-being of the critically ill population are indisputable [13]. However, inconsistent oral health care practice has been reported among critical care nurses in the intensive care setting [14].

Care-dependent elderly living in long-term care facilities is entitled to receive adequate oral health care and registered nurses are one of the key professional groups responsible for this task [15]. Home care nursing guidelines are clear about daily oral health care as a primary component of activities of daily living care [16]. An oral health care program that included home care nurses providing care/daily oral health care to older people has been shown to not only improve nurses’ knowledge and attitude but also the oral health of older adults [16]. Therefore, oral health care is an important nursing responsibility, and teaching nurses appropriate skills to support oral health is a necessary prerequisite to implementing best nursing practices.

Despite the importance of oral health, competence in oral health and attitudes toward oral health care is inadequate in nursing care. Oral health is one most neglected nursing care activities [17]. In India, Philip and associates showed that the nurses’ knowledge is poor with specific deficits in common medications that affect oral health and care of dentures as well as inconsistencies in oral health assessment and care protocols [18]. To complicate this issue further, inadequate oral health content in entry-level nursing curricula is a well-known issue [19]. These demonstrated a great need to improve nurses’ knowledge and skills to provide standardized and effective oral health care in nursing education [20].

Oral health education can improve nursing students’ knowledge, skills, and attitudes toward oral health [17]. In addition to knowledge, practice experiences are also essential to build confidence, motivation, and skills in oral health [21]. Oral health guidance requires a series of operation steps and equipment; thus, collaborative interprofessional education that engages students in oral health instruction demonstrations can help to increase oral health literacy and knowledge [22]. To overcome the difficulty of traditional teaching methods, limited skill simulation exercises have been piloted [23]. However, a single dose of simulation training was found to have a minimal impact on critical care nurses’ skill scores related to the recommended oral health care practices, supporting the need for regularly repeated training approaches [24].

Virtual reality (VR) is capable of achieving a series of practical exercises, thus, an effective method for learning operational skills [25, 26]. Emerging studies have uncovered some benefits of VR in undergraduate nursing education, especially in effective nurse skill acquisition, such as chemotherapy administration, and intravenous catheter insertion [27, 28]. However, despite VR being increasingly used in nursing education, this technology remains a relatively new experience for many nursing students. A prior study showed that a VR training system had a positive effect on dental health students’ [29]. The objective of this study was to evaluate the use of the VR-based geriatric oral health training system on knowledge, attitude, self-efficacy, and intentions in providing oral healthcare to older adults among undergraduate nursing students. Such technology-facilitated tools for training nursing students in effective oral health support for individuals needing assistance is an example of an oral health promotion tool currently being focused on by the National Institutes of Health’s Funding Opportunity Announcement, “Harnessing Technologies to Support Oral Health Promotion and Management Outside the Dental Setting [30]. The aims of this study were to evaluate the effects and satisfaction of virtual reality-based education on geriatric oral health for nursing students.

## Methods

### Design

The Consolidated Standards of Reporting Trials (CONSORT) checklist for randomized control trial was adopted to report the results of this study. A parallel-group pilot randomized controlled design was used in this study. The study was conducted at the Department of Nursing, Kaohsiung Medical University, Taiwan. The study has been registered at www.clinicaltrials.gov (registration number: NCT05248542; registration date 21/02/2022).

### Participants

The study participants included undergraduate freshman, sophomore, and junior nursing students who volunteered to participate in this study. Exclusion criteria included students with known negative effects, such as nausea, vomiting, etc., from using VR devices and those under 20 years of age who had not obtained the consent of a legal representative to participate in this study. All nursing students were initially enrolled face-to-face to provide informed consent.

The sample size calculation was set up for two-tailed repeated measures ANOVA between factors, an alpha level of 0.05, power of 80%, three times measurements, correlation of 0.7, and effect size f of 0.375 which was referenced the effect sizes Cohen’s d ranged 0.75 to 1.35 in the previous study by G*Power 3.1.9.4 software [29]. The optimal sample size was 40. A total of 52 students were recruited for this study (26 per the experimental and the control group).

The first author generated the random allocation sequence by using the online randomization number generator (https://lab.25sprout.com/nrprnd/). The students were randomly assigned and unblinded to the experimental and control group. Simple randomization with a 1:1 allocation ratio was used.

### Intervention

**Fig. 1:**
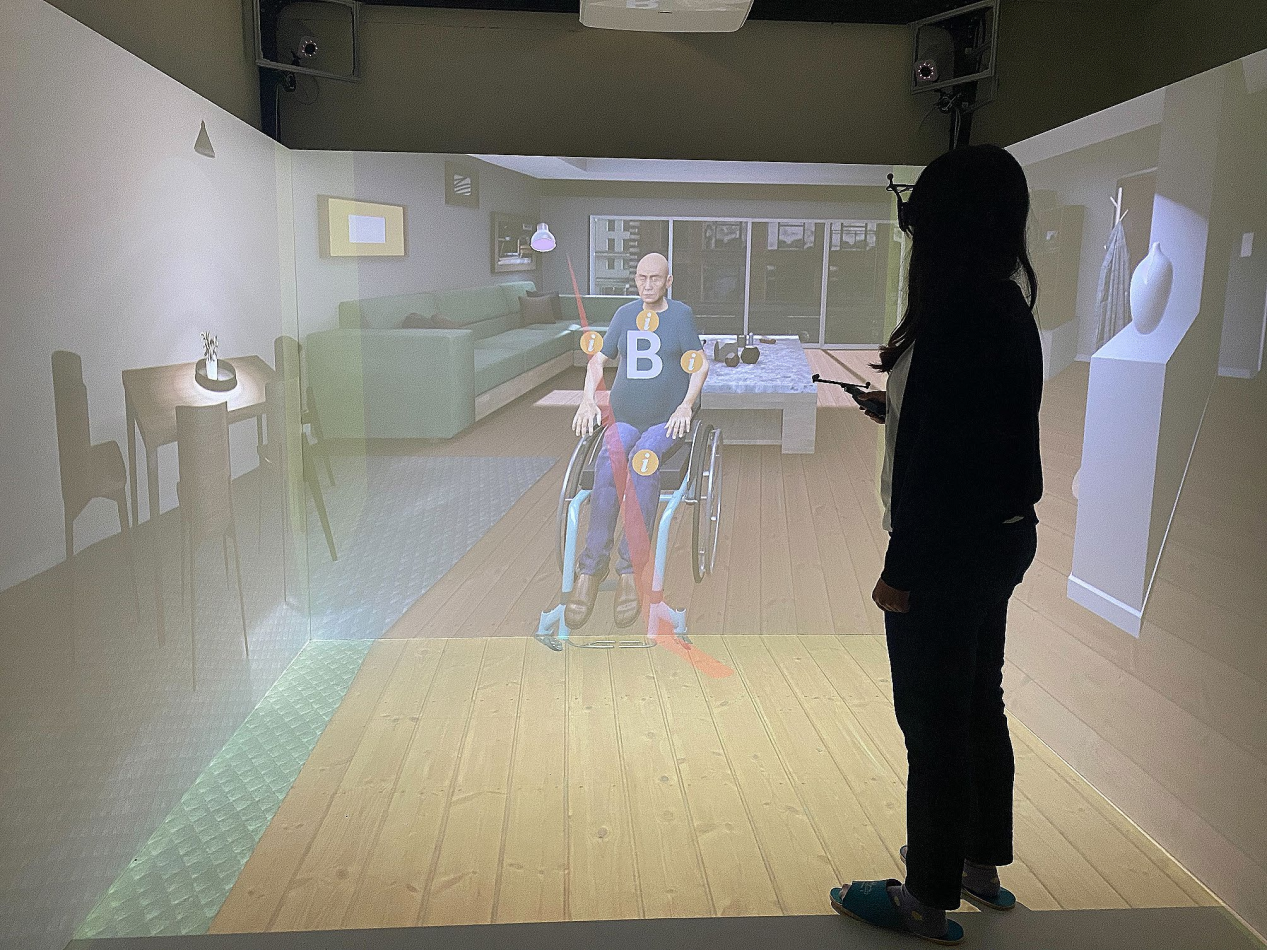
The virtual reality-based simulation education on geriatric oral hygiene care

The VR Oral Health Care Learning System (Pvix VR, eped Inc., Taiwan) was equipped with an optical portable wearable device that utilized positioning motion recognition technology and a built-in virtual interactive software to assist in the self-training (https://www.youtube.com/watch?v=GpVnl8gWKd4). Each participant in the experimental group received a 30-minute individualized VR-based oral health care simulation training in a dedicated oral skills training room. First, the participant was instructed to wear the 3D glasses with remote control (5 min) followed by the VR-based simulation training. The participants practiced choosing step actions by using the remote control according to the virtual screen whereas a series of speech and text system instructions were presented (Fig. 1).

The contents of VR-based training on geriatric oral health care used in this study for nursing students were based on the training of dental health students [29]. The VR-based simulation training on geriatric oral health care section started with a system guide explaining the VR-based older adult’s physical health and oral health (2 min) followed by a scene of a virtual old man sitting in a wheelchair next to a bathroom sink. The old man had partial dentures, some natural teeth, and a dry mouth. The participant was evaluated to demonstrate the following competencies: (1) Greet the old man and explain the reason for providing oral health care (2 min); (2) Inspect the virtual physical environment for safety and adequacy to perform mouth care, for example, unobstructed space, handrail, dry floor, adequate sink height, accessibility of dental cleaning tools, proper sitting posture, etc. (2 min); (3) Able to choose and use proper denture cleaning products to assist in cleaning dentures (3–5 min); (4) Able to assist in cleaning of natural teeth (5–7 min) including choosing and using the proper teeth cleaning products and instruments as well as moisturizing oral rinses for dry mouth (2 min). After completing the VR-based simulation training, each participant was required to correctly answer four questions designed to evaluate (1) the correct storage method for dentures, (2) the proper selection of interdental cleaning aids, (3) the identification of suitable toothpaste with optimal fluoride concentration, and (4) optimal frequency of regular dental check-ups. If the participant selected an incorrect answer, the learning system would generate feedback to guide the participant to the correct answer (3–5 min). The same oral health care training system and teaching content were repeated the second time two weeks later.

To accurately simulate the current lack of standardized geriatric oral health learning content in nursing curricula, the control group only received repeated questionnaires.

### Instruments

All participants received an online questionnaire at study enrollment and conclusion. The questionnaire contained 37 items to evaluate the participants’ knowledge, attitudes, and self-efficacyof geriatric oral health as well as their intention to assist in oral health care for older adults. This questionnaire was adapted from a previous study [26]. The face validity was established by four experts, including a professor and an experienced dentist in the college of dentistry, an assistant professor of nursing with a master’s degree in dental health, a nursing director of a nursing home, and a professor of public health with expertise in nursing education, health promotion, and health behaviors. Demographic information, including age, sex, academic year, as well as prior VR game experience and prior oral health learning experience were obtained from all participants. Participants in the experimental group also completed the System Usability Scale (SUS) questionnaire to evaluate their satisfaction with the VR Oral Health Care Learning System.

#### Knowledge of geriatric oral health scale

The questionnaire contained 10 items to evaluate the participants’ geriatric oral health knowledge (Supplementary material 1). Each item had three response options: true, false, and unknown. Each item was assigned one point for the correct response and no point for the incorrect or unknown responses. The total score ranged from 0 to 10 and a higher score indicated a higher level of geriatric oral health knowledge. The Kuder-Richardson Formula 20 was 0.57 for the knowledge scale in this study.

#### Attitudes towards geriatric oral health scale

The attitudes towards geriatric oral health scale included seven items (Supplementary material 1). Each item was scored on a five-point Likert scale ranging from 1 (strongly disagree) to 5 (strongly agree). Reverse items were scored from 5 (strongly disagree) to 1 (strongly agree). The total score ranged from 7 to 35 and a higher score indicated a positive attitude toward geriatric oral health. Cronbach’s alpha was 0.7 for the attitudinal scale.

#### Self-efficacy of geriatric oral health scale

The self-efficacy of geriatric oral health scale included ten items (Supplementary material 1). Each item was scored on a five-point Likert scale ranging from 1 (strongly disagree) to 5 (strongly agree) and the higher score indicated a higher level of geriatric oral health self-efficacy. The total score ranged from 10 to 50. Cronbach’s alpha was 0.84 for the self-efficacy scale.

### Intention to assist oral health care for older adult scale

The behavioral intention to assist oral health care for older adult scale included ten items (Supplementary material 1). Each item was scored on a five-point Likert scale, ranging from 1 (strongly disagree) to 5 (strongly agree), with a higher score indicating a higher level of intention to assist oral health care for the older adult. The total score ranged from 10 to 50. Cronbach’s alpha for the intention to assist oral health care for older adult scale was 0.91.

### System usability of VR-based learning system

The participants’ satisfaction with the VR-based learning system for geriatric oral health training was evaluated using the Chinese version of the System Usability Scale. The System Usability Scale is a simple, subjective assessment to evaluate the effectiveness, efficiency, and satisfaction of system usability [31]. The Chinese version of the System Usability Scale included ten items and has acceptable sensitivity, reliability, and validity [29]. The score calculation was based on a specific formula [32]. The total score ranged from 0 to 100 with a score above 68 as a benchmark of an average experience and a score above 80 as a good experience [33].

### Procedure

Participants received a consent form that included the purpose, design, activities, and benefits of this study. Written informed consent to participate in this study was received before data collection began. The VR-based learning system was used by the participants in the experimental group, thus, participants and researchers were not blinded to the group assignments.

At baseline (Time 0), each participant received a research code and hyperlink to Google forms to complete demographic information and study questionnaire. The participants assigned to the experimental group were instructed to schedule two sessions of the VR-based training - at two (Time 1) and fourth weeks (Time 2) after baseline data collection. Immediately after each VR-based training session, each participant was asked to complete the post-test questionnaire. The participants in the control group were asked to complete the post-test questionnaires through hyperlinks two and four weeks after the baseline session.

### Data analysis

Data analyses were performed using IBM SPSS Statistics (Version 26.0) and Stata 13.1. The baseline data between the experimental and control groups were compared using independent *t*-tests and Chi-square tests to examine the homogeneity. Independent *t*-tests were used to assess the differences in knowledge, attitudes, self-efficacy, and intention to assist oral health care between the two groups at baseline, Time 1, and Time 2. A generalized estimating equation (GEE) of longitudinal data analysis was employed to analyze the effects of the VR-based intervention on participants’ knowledge, attitudes, self-efficacy, and intention to assist oral health care, taking into consideration the factors, including gender, academic year (sophomore, junior, etc.), previous experience of VR games, and previous oral health learning, that could explain or modify the relationship between intervention and the outcome variables. The alpha level for statistical significance was set at 0.05. Effect size estimates were calculated for all mean differences using Cohen’s *d*. For participants in the experimental group, a simple linear regression was used to explore different knowledge, attitude, and self-efficacy scores between the baseline and Time 1 to predict the change in behavioral intention scores between the baseline and Time 2. The satisfaction score of the VR Oral Health Care Learning System was analyzed by mean scores and standard differences using descriptive statistics.

## Results

### Participant recruitment and retention

The Consolidated Standards of Reporting Trials (CONSORT) checklist for randomized control trials was adopted to report the results of this study. Between March 2021 and November 2021, 52 participants were assessed for eligibility, with two participants excluded because they did not fill in the initial questionnaires, resulting in 50 participants being randomized (the experimental group was 25 and the control group was 25). No participant withdrew from the study and all participants completed the study protocol (Supplementary material 2).

### Baseline characteristics and mean scores of the outcome measures from baseline to 4 weeks follow-up between the experimental group and control group

Participants’ demographic characteristics and previous learning experiences are presented in Table 1. The mean age of the participants was 21.30 ± 1.15 years. The majority of the participants were female (80%), juniors in the undergraduate nursing program (68.0%), had no prior VR gaming experience (80.0%) and had no prior experience in oral health care learning (74.0%). Participants’ demographics, prior VR gaming, and prior oral health care learning experience were not statistically different between the two groups (all *p* > 0.05).

**Table 1:** Nursing students’ demographics and prior learning experiences

	Total	Experimental (*n* = 25)	Control (*n* = 25)	t/χ^2^	p
	n (%)	n (%)	n (%)		
**Age** (years, Mean ± SD)	21.30 ± 1.15	21.3 ± 1.10	21.3 ± 1.22	-0.12	0.903
**Sex**				0.50	0.480
Male	10 (20.0)	4 (16.0)	6 (24.0)		
Female	40 (80.0)	21 (84.0)	19 (76.0)		
**Academic year**				3.33	0.190
First-year	6 (12.0)	2 (8.0)	4 (16.0)		
Second year	10 (20.0)	3 (12.0)	7 (28.0)		
Third year	34 (68.0)	20 (80.0)	14 (56.0)		
**Experience with VR game**				0.00	1.000
No	40 (80.0)	20 (80.0)	20 (80.0)		
Yes	10 (20.0)	5 (20.0)	5 (20.0)		
**Experience in oral hygiene learning**				0.10	0.747
No	37 (74.0)	19 (76.0)	18 (72.0)		
Yes	12 (26.0)	6 (24.0)	7 (28.0)		

SD, standard deviation

**Fig. 2:**
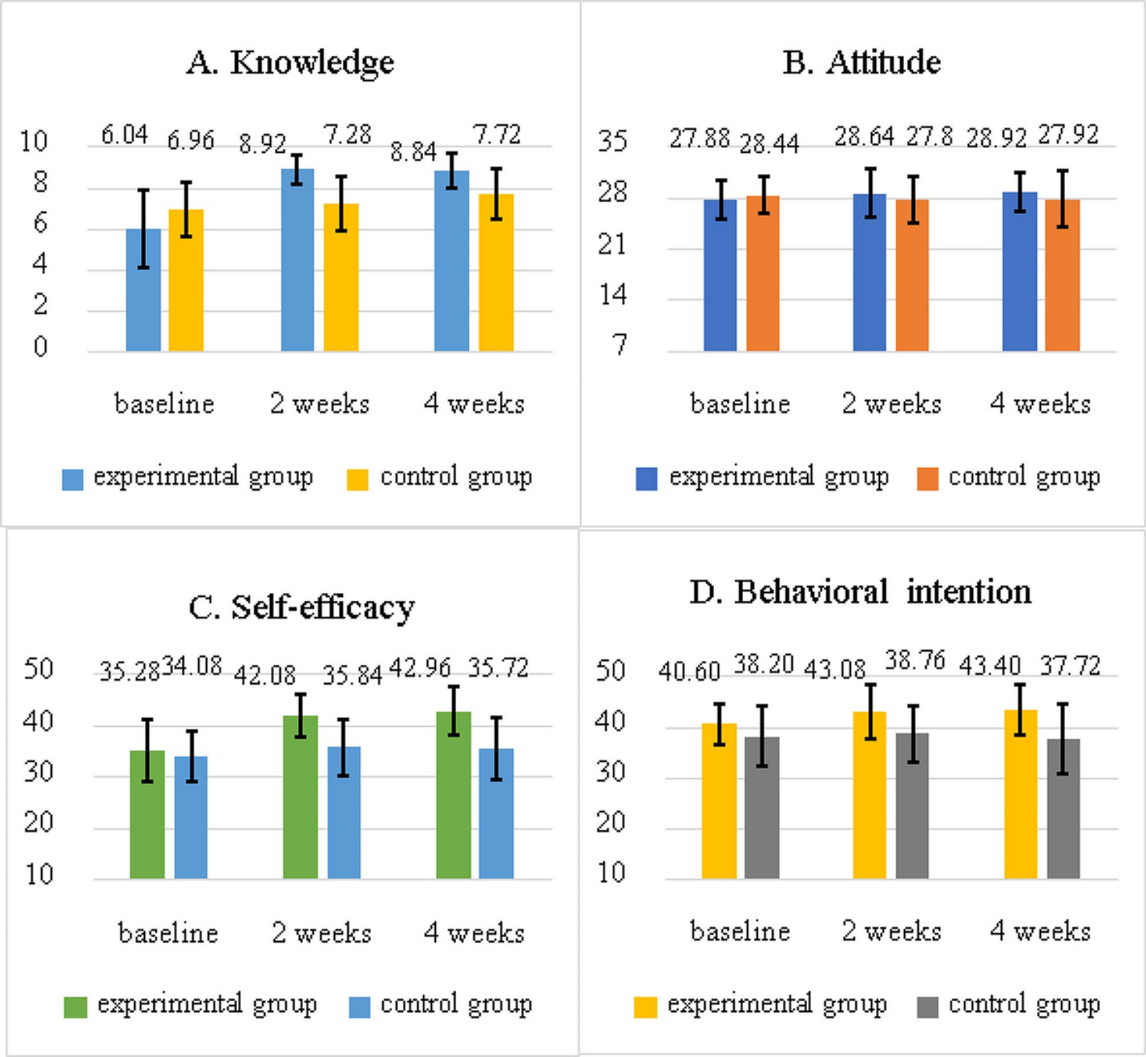
The mean scores of the outcome measures from baseline to 4 weeks follow-up between the experimental group and control group

For all participants, the mean baseline geriatric oral health knowledge score was 6.50 ± 1.71, the attitude towards geriatric oral health score was 28.16 ± 2.63, the self-efficacy of geriatric oral health score was 34.68 ± 5.52, and intention to assist oral health care for the older adult score was 39.40 ± 5.19. There was no ceiling or floor effect on the questionnaire. The proportion of respondents scoring the highest (ceiling) or lowest (floor) possible scores was under 15% (Appendix 3). At baseline, all mean scores of knowledge, attitudes, and self-efficacy of geriatric oral health as well as the intention to assist oral health care for older adults were not statistically different between the control and experimental groups (all *p* > 0.05) (Table 2). There were significantly different knowledge, self-efficacy, and behavioral intention at both Time 1 and Time 2 between the experimental and control groups (all *p* < 0.05) (Fig. 2).

**Table 2:** Mean scores of outcome measures from baseline to 4 weeks follow-up between the experimental group and control group

Outcomes	Total	Experimental (*n* = 25)	Control (*n* = 25)	t	p value
	n (%)	n (%)	n (%)		
Knowledge T0	6.50 ± 1.71	6.04 ± 1.90	6.96 ± 1.37	-1.96	0.056
Knowledge T1	8.10 ± 1.34	8.92 ± 0.70	7.28 ± 1.34	5.42	< 0.001
Knowledge T2	8.28 ± 1.18	8.84 ± 0.85	7.72 ± 1.21	3.79	< 0.001
Attitude T0	28.16 ± 2.63	27.88 ± 2.65	28.44 ± 2.63	0.75	0.457
Attitude T1	28.22 ± 3.26	28.64 ± 3.29	27.80 ± 3.24	0.91	0.368
Attitude T2	28.42 ± 3.23	28.92 ± 2.60	27.92 ± 3.75	1.10	0.279
Self-efficacy T0	34.68 ± 5.52	35.28 ± 6.15	34.08 ± 4.86	0.77	0.448
Self-efficacy T1	38.96 ± 5.83	42.08 ± 4.19	35.84 ± 5.61	0.27	< 0.001
Self-efficacy T2	39.34 ± 6.55	42.96 ± 4.82	35.72 ± 6.09	0.52	< 0.001
Behavioral Intention T0	39.40 ± 5.19	40.60 ± 4.16	38.20 ± 5.88	1.67	0.103
Behavioral Intention T1	40.92 ± 5.71	43.08 ± 5.18	38.76 ± 5.49	2.86	0.006
Behavioral Intention T2	40.56 ± 6.53	43.40 ± 5.00	37.72 ± 6.74	3.39	0.001

T0: baseline, T1: 2 weeks follow up, T2: 4 weeks follow up

### Intervention effect on knowledge, attitudes, and self-efficacy of geriatric oral health and intention to assist oral health care for older adult

The results of the GEE analysis after controlling for gender, academic year, the experience of VR game, and the experience of oral health learning, showing the association between the VR-based simulation training on geriatric oral health care for nursing students are presented in Table 3. Compared to the participants in the control group, those in the experimental group had significantly greater improvements in geriatric oral health care knowledge [*β*: 2.56, 95% CI: 1.05, 3.03; *p* < 0.001; effect size: 1.15], and self-efficacy [*β*: 5.04, 95% CI: 1.70, 8.38; *p* = 0.003; effect size: 0.82] after the first round of training. The higher geriatric oral health care knowledge training [*β*: 2.04, 95% CI: 1.57, 3.55; *p* < 0.001; effect size: 1.40], attitudes [*β*: 1.56, 95% CI: 0.25, 2.88; *p* = 0.020; effect size: 0.64], self-efficacy [*β*: 6.04, 95% CI: 2.93, 9.16; *p* < 0.001; effect size: 1.05], and intention to assist oral health care for older adult scores [*β*: 3.28, 95% CI: 0.32, 6.24; *p* = 0.030; effect size: 0.60] among participants in the experimental group than those in the control group persisted after the second round of training.

**Table 3:** Effects of the VR-based intervention on outcome variables, taking into consideration the factors that could explain or modify this relationship using generalized estimating equation (GEE) model

Parameter	β	S.E.	95% C.I.		p value	E.S.
Knowledge^a^						
Intercept	5.97	0.58	4.84	7.10	< 0.001	
Group	-1.06	0.49	-1.96	-0.17	0.020	
Time 1	0.32	0.33	-0.32	0.96	0.330	
Time 2	0.76	0.34	0.09	1.43	0.026	
Group × Time 1	2.56	0.51	1.57	3.55	< 0.001	1.15
Group × Time 2	2.04	0.51	1.05	3.03	< 0.001	1.40
Attitude^a^						
Intercept	25.62	1.54	22.60	28.65	< 0.001	
Group	-0.93	0.75	-2.40	0.54	0.213	
Time 1	-0.64	0.43	-1.48	0.20	0.137	
Time 2	-0.52	0.49	-1.48	0.44	0.286	
Group × Time 1	1.40	0.75	-0.07	2.87	0.062	0.52
Group × Time 2	1.56	0.67	0.25	2.88	0.020	0.64
Self-efficacy^a^						
Intercept	31.13	2.36	26.50	35.76	< 0.001	
Group	0.46	1.52	-2.51	3.44	0.760	
Time 1	1.76	0.87	0.06	3.46	0.043	
Time 2	1.64	1.05	-0.42	3.70	0.119	
Group × Time 1	5.04	1.70	1.70	8.38	0.003	0.82
Group × Time 2	6.04	1.59	2.93	9.16	< 0.001	1.05
Behavioral intention^a^						
Intercept	37.75	2.15	33.52	41.97	< 0.001	
Group	2.39	1.49	-0.54	5.32	0.109	
Time 1	0.56	0.84	-1.08	2.20	0.504	
Time 2	-0.48	1.16	-2.75	1.79	0.679	
Group × Time 1	1.92	1.24	-0.52	4.36	0.123	0.43
Group × Time 2	3.28	1.51	0.32	6.24	0.030	0.60

C.I., confidence intervals

S.E., standard errors

E.S., Effect size: calculated by the mean change scores between pre- and post-test measurements between the two groups

Time 1: 2 weeks follow up, Time 2: 4 weeks follow up

^a^All GEE models were after controlling for gender, academic year, experience of VR game and, experience of oral hygiene learning

For participants in the experimental group, the change score of oral health care knowledge between Time 0 and Time 1 had a significant impact on the change scores of behavioral intentions between Time 0 and Time 2 [*β*: 1.27, 95% CI: 0.33, 2.20; *p* = 0.010]. The change scores of attitudes [*β*: -0.13, 95% CI: -0.81, 0.55; *p* = 0.690] and self-efficacy [*β*: 0.05, 95% CI: -0.23, 0.33; *p* = 0.719] between Time 0 and Time 1 did not have a significant impact on change scores of behavioral intentions between Time 0 and Time 2.

### Satisfaction of VR-based learning system on geriatric oral health

The mean satisfaction score of the VR Oral Health Care Learning System among 25 participants was 79.1 ± 10.36 (range: 57.5–100). Twenty-two (88%) and 11 (44%) participants rated 68 and 80 points or over on their satisfaction with the VR Oral Health Care Learning System, respectively. The satisfaction score of the VR Oral Health Care Learning System was not different between those with and without previous VR game experience (*n* = 5, mean satisfaction score = 81.00 ± 4.87; *n* = 20, mean satisfaction score 78.63 ± 11.37, *t* = 0.45, *p* = 0.656, respectively).

## Discussion

In this age of digital transformation, the VR system has been used as an individual self-learning training tool [29, 34]. To the best of the authors’ knowledge, the current study is the first to explore the multiple domains of the effectiveness of a VR-based learning system on geriatric oral health care in undergraduate nursing students. The results of this study showed that two rounds of VR-based training delivered over 4 weeks significantly improved the nursing students’ knowledge, attitude, and self-efficacy of geriatric oral health and intention to assist oral health care for older adults. The improvement in attitudes towards geriatric oral health care and intention to assist oral health care for older adults was only observed in the experimental group after the second round of VR-based training, suggesting repeated teaching/learning interventions are needed to enhance the effectiveness of oral health training. The findings of the current study also showed that the majority of nursing students were satisfied with the VR Oral Health Care Learning System on geriatric oral health. The findings of the current study support that VR-based training is an effective and accepted learning strategy to improve nursing students’ oral health care practices.

Previous studies have indicated that education can improve healthcare professional undergraduate students’ oral health knowledge and attitudes [35, 36]. For example, a competency-based curriculum in oral health care can improve the knowledge and positive attitudes toward oral health care among undergraduate dental therapy and oral health students [37]. An interprofessional education curriculum with an illustrated booklet and toothbrushing and flossing technique presentation by dental and dental health faculty can improve oral health literacy in dentistry, medicine, and nursing students [21]. Even though a dental undergraduate curricula can influence students’ knowledge and positive attitudes, its influence on students’ behavioral modifications is limited [37]. It has been reported that curricula with traditional lectures and face-to-face presentation teaching methods without practical experience may limit the improvement intention to assist oral health care for older adults [17]. In the current study, the VR-based learning system is effective in improving nursing students’ attitudes toward geriatric oral health care and intention to assist oral health care for older adult. The participants in this study included nursing students from the first to third years, implying potential differences in their learning progression. After adjusting gender, academic year, experience of VR game and experience of oral hygiene learning, VR-based simulation training on geriatric oral health improved knowledge and self-efficacy after the first round of training. The knowledge improvement predicts improvement in behavioral intention to assist oral health care for older adults after the second round of training.

Our prior work showed that students in an oral health program had a large improvement effect in knowledge, self-efficacy of oral health care, and intention to assist in oral health care behaviors and a near-large enhancement effect in their attitude toward oral health care after two rounds of VR-based oral health care training [29]. The current study indicated that nursing students only get a large improvement effect in knowledge and self-efficacy and a medium enhancement effect in their attitude toward oral health care and intention to assist in oral health care behaviors after two rounds of VR-based oral health care training. Attitude and intention were the most important determinants to engage in oral health care behavioral adoption among nurses working in intensive care units [14]. A systematic review indicated that undergraduate nursing students had limited oral health knowledge and varying attitudes toward providing oral health care [21]. Findings from these studies and the current study showed the importance of improving nurses ’ attitudes and intention to assist oral health care in nursing practice and the VR-based learning system can be such technology integrated into the nursing curriculum to enhance students’ positive attitudes and behavioral intention.

Medical students preferred the VR-based method to learn nausea and vomiting management in palliative care and oncology settings [38]. Medical students enjoyed the learning experience about VR learning environments in obstetrics and were enhanced in their satisfaction and confidence of conceptualize fetal lies and presentation [39]. In addition, a report from a recent systematic review showed that medical education training using VR-based head-mounted devices was perceived as engaging [40], however, limited studies support the use of these devices for effective knowledge and skills training in the fields of surgery and anatomy [40]. For the current study, VR-based head-mounted devices were effective for knowledge, attitude, self-efficacy, and intention in the fields of geriatric oral health care training for undergraduate nursing students. In our small study, the undergraduate nursing students’ satisfaction with the training system does not appear to be influenced by gender or video game experience. The utility of VR-based practical exercises to teach operational skills should be further explored. Periodontal disease can be prevented and treated by relagular plaque cleaning [8]. The VR-based head-mounted devices have applied on patients with mild cognitive impairment to improve the cognition and motor function [41]. The future study could evaluate the use of a VR-based Oral Health Care Learning System to maintain the patient’s daily oral health among patients with mild cognitive impairment.

The current study has some limitations. First, blinding was unable to be implemented in this study as the informed consent specified the procedures involved for participants in the experimental and control groups. Secondly, the generalization of findings to other students or other institutions should be carefully considered. Because students were recruited by convenient sampling, the number of students participating in this study was only about 25% of the total number of nursing students in the three academic years. Third, the goal of this study was to evaluate the effects and satisfaction of a VR-based geriatric oral health training system. In view of the fact that the traditional lecture courses for Taiwan nursing students do not include this oral health care content. Therefore, it is suggested that further study can compare the effectiveness of VR situational simulation training and traditional classroom teaching.

## Conclusion

This study has shown that a VR-based learning system was effective to improve the knowledge, attitudes, and self-efficacy of geriatric oral health and intention to assist oral health care for the older adult among undergraduate nursing students. A single dose of training may be sufficient in improving nursing students’ knowledge and self-efficacy of geriatric oral health. However, repeated training at least twice may be minimally required to improve nursing students’ attitudes toward geriatric oral health and intention to assist oral health care for older adults. This VR-based learning system for geriatric oral health has the potential to be used as a tool in nursing undergraduate education to provide the important training known to be lacking and/or deficient in the current curriculum.

### Electronic supplementary material

Below is the link to the electronic supplementary material.

Supplementary Material 1

Supplementary Material 2

Supplementary Material 3

## Data Availability

The datasets used and/or analysed during the current study available from the corresponding author on reasonable request.

## References

[1] Tonetti MS, Bottenberg P, Conrads G, Eickholz P, Heasman P, Huysmans MC, Lopez R, Madianos P, Muller F, Needleman I, Nyvad B, Preshaw PM, Pretty I, Renvert S, Schwendicke F, Trombelli L, van der Putten GJ, Vanobbergen J, West N, Young A, Paris S (2017). Dental caries and periodontal diseases in the ageing population: call to action to protect and enhance oral health and well-being as an essential component of healthy ageing – Consensus report of group 4 of the joint EFP/ORCA workshop on the boundaries between caries and periodontal diseases. J Clin Periodontol.

[2] Hughes MJ, Gazmararian JA (2015). The relationship between income and oral health among people with intellectual disabilities: a global perspective. Spec Care Dentist.

[3] Albani V, Nishio K, Ito T, Kotronia E, Moynihan P, Robinson L, Hanratty B, Kingston A, Abe Y, Takayama M, Iinuma T, Arai Y, Ramsay SE (2021). Associations of poor oral health with frailty and physical functioning in the oldest old: results from two studies in England and Japan. BMC Geriatr.

[4] Algra Y, Haverkort E, Kok W, Etten-Jamaludin FV, Schoot LV, Hollaar V, Naumann E, Schueren MV, Jerković-Ćosić K (2021). The association between malnutrition and oral health in older people: a systematic review. Nutrients.

[5] Kotronia E, Brown H, Papacosta AO, Lennon LT, Weyant RJ, Whincup PH, Wannamethee SG, Ramsay SE (2021). Oral health and all-cause, cardiovascular disease, and respiratory mortality in older people in the UK and USA. Sci Rep.

[6] Laouali N, Fatouhi E, Aguayo D, Balkau G, Boutron-Ruault B, Bonnet MC, F., Fagherazzi G. Type 2 diabetes and its characteristics are associated with poor oral health: findings from 60,590 senior women from the E3N study. BMC Oral Health. 2021;21(1). 10.1186/s12903-021-01679-w. 10.1186/s12903-021-01679-w PMC8220760 34162373

[7] Scannapieco FA (2021). Poor oral health in the etiology and prevention of aspiration pneumonia. Dental Clin N Am.

[8] Ramírez JH, Arce RM, Contreras A (2011). Periodontal treatment effects on endothelial function and cardiovascular disease biomarkers in subjects with chronic periodontitis: protocol for a randomized clinical trial. Trials.

[9] van de Rijt LJM, Stoop CC, Weijenberg RAF, de Vries R, Feast AR, Sampson EL, Lobbezoo F (2020). The influence of oral health factors on the quality of life in older people: a systematic review. Gerontologist.

[10] Coll PP, Lindsay A, Meng J, Gopalakrishna A, Raghavendra S, Bysani P, O’Brien D (2020). The prevention of infections in older adults: oral health. J Am Geriatr Soc.

[11] Hazara R (2020). Oral health in older adults. Br J Community Nurs.

[12] Bonetti D, Hampson V, Queen K, Kirk D, Clarkson J, Young L (2015). Improving oral hygiene for patients. Nurs Standard.

[13] Berry AM, Davidson PM (2006). Beyond comfort: oral hygiene as a critical nursing activity in the intensive care unit. Intensive Crit Care Nurs.

[14] Tanguay A, Lemay S, Reeves I, Gosselin E, St-Cyr-Tribbkle D (2020). Factors influencing oral care in intubated intensive care patients. Nurs Crit Care.

[15] Samson H, Iversen MM, Strand GV (2010). Oral care training in the basic education of care professionals. Gerodontology.

[16] Weening-Verbree LF, Schuller AA, Zuidema SU, Hobbelen JSM. Evaluation of an oral care program to improve oral health of home-dwelling older people. Int J Environ Res Piblic Health. 2022;19(12). 10.3390/ijerph19127251. Article 7251. 10.3390/ijerph19127251 PMC9223830 35742500

[17] Lewis A, Edwards S, Whiting G, Donnelly F (2018). Evaluating student learning outcomes in oral health knowledge and skills. J Clin Nurs.

[18] Philip P, Villarosa A, Gopinath A, Elizabeth C, Norman G, Geroge A (2019). Oral health knowledge, attitude and practices among nurses in a tertiary care hospital in Bangalore, India: a cross-sectional survey. Contemp Nurse.

[19] Ahmad MS, Abuzar MA, Razak IA, Rahman SA, Borromeo GL (2021). Oral health education in the undergraduate nursing curriculum of Australian and Malaysian institutions. Eur J Dent Educ.

[20] Pai M, Ribot B, Tane H, Murray J (2016). A study of periodontal disease awareness amongst third-year nursing students. Contemp Nurse.

[21] Bhagat V, Hoang H, Crocombe LA, Goldberg LR (2020). Incorporating oral health care education in undergraduate nursing curricula - a systematic review. BMC Nurs.

[22] Farokhi MR, Muck A, Lozano-Pineda J, Boone SL, Worabo H (2018). Using interprofessional education to promote oral health literacy in a faculty-student collaborative practice. J Dent Educ.

[23] Golinveaux J, Gerbert B, Cheng J, Duderstadt K, Alkon A, Mullen S, Lin B, Miller A, Zhan L (2013). Oral health education for pediatric nurse practitioner students. J Dent Educ.

[24] Jansson MM, Syrjälä HP, Ohtonen PP, Meriläinen MH, Kyngäs HA, Ala-Kokko TI (2017). Effects of simulation education on oral care practices - a randomized controlled trial. Nurs Crit Care.

[25] Aksoy E. Comparing the effects on learning outcomes of tablet-based and virtual reality–based serious gaming modules for basic life support training: randomized trial. JMIR Serious Games. 2019;7(2). 10.2196/13442. Article e13442. 10.2196/13442 PMC6660122 31042153

[26] Berg H, Steinsbekk A. Is individual practice in an immersive and interactive virtual reality application non-inferior to practicing with traditional equipment in learning systematic clinical observation? A randomized controlled trial. BMC Med Educ. 2020;20(1). 10.1186/s12909-020-02030-7. 10.1186/s12909-020-02030-7 PMC7181571 32326948

[27] Chan H-Y, Chang H-C, Huang T-W (2021). Virtual reality teaching in chemotherapy administration: Randomised controlled trial. J Clin Nurs.

[28] İsmailoğlua EG, Orkun N, Eşer İ, Zaybak A (2020). Comparison of the effectiveness of the virtual simulator and video-assisted teaching on intravenous catheter insertion skills and self-confidence: a quasi-experimental study. Nurse Educ Today.

[29] Chang A-H, Lin P-C, Lin P-C, Lin Y-C, Kabasawa Y, Lin C-Y, Huang H-L (2022). Effectiveness of virtual reality-based training on oral healthcare for disabled elderly persons: a randomized controlled trial. J Personalized Med.

[30] NIH Central Resource for Grants and Funding Information. (2022). Harnessing Technologies to Support Oral Health Promotion and Management Outside the Dental Setting (UG3/UH3 Clinical Trial Required) Available: https://grants.nih.gov/grants/guide/rfa-files/RFA-DE-23-009.html [access 2022-10-28].

[31] Lewis JR, Sauro J (2018). Item benchmarks for the System Usability Scale. J Usability Stud.

[32] Wang Y, Lei T, Liu X (2020). Chinese system usability scale: translation, revision, psychological measurement. Int J Human–Computer Interact.

[33] Guerci J. (2020). Easily calculate SUS score. A quick tutorial to automate this task and save time. [Online]. Available: https://uxplanet.org/easily-calculate-sus-score-a464d753e5aa [accessed 2022-07-12].

[34] Fairén M, Moyés J, Insa E (2020). VR4Health: personalized teaching and learning anatomy using VR. J Med Syst.

[35] de Sam Lazaro SL, Karger TR, Despres BR, McPherson RC (2022). An approach to developing oral health knowledge for allied health students. J Dent Educ.

[36] Sabounchi SS, Sabounchi SS, Safari M (2019). Knowledge and attitude of midwifery students on oral health care. Dentistry J.

[37] Singh S (2017). Can undergraduate student learning in prevention influence oral health self-care practices? A report from a South African university. Int J Dental Hygiene.

[38] Taubert M, Webber L, Hamilton T, Carr M, Harvey M (2019). Virtual reality videos used in undergraduate palliative and oncology medical teaching: results of a pilot study. BMJ Support Palliat Care.

[39] Kane D, Ryan G, Mangina E, McAuliffe FM (2022). A randomized control trial of a virtual reality learning environment in obstetric medical student teaching. Int J Med Informatics.

[40] Barteit S, Lanfermann L, Bärnighausen T, Neuhann F, Beiersmann C. Augmented, mixed, and virtual reality-based head-mounted devices for medical education: systematic review. JMIR Serious Games. 2021;9(3). 10.2196/29080. Article e29080. 10.2196/29080 PMC8299342 34255668

[41] Ren Y, Wang Q, Liu H, Wang G, Lu A (2024). Effects of immersive and non-immersive virtual reality-based rehabilitation training on cognition, motor function, and daily functioning in patients with mild cognitive impairment or dementia: a systematic review and meta-analysis. Clin Rehabil.

