# Supplementary material for: Virtual reality-based simulation learning on geriatric oral health care for nursing students: a pilot study
Source: BMC Oral Health. 2024 May 28;24:627. doi: 10.1186/s12903-024-04249-y (PMC11134768; doi:10.1186/s12903-024-04249-y)
Supplement: Supplementary file 1 — Supplementary Material 1 [file 12903_2024_4249_MOESM1_ESM.docx]

Appendix 1. Questionnaire

Table S1. Items and appropriate answers of knowledge of geriatric oral health scale

| Item | Appropriate answer |
| --- | --- |
| 1. Dentures should be cleaned with toothpaste every day. | False |
| 2. Dentures can be soaked in antibacterial denture cleanser overnight. | False |
| 3. Hot water must not be used to clean dentures. | True |
| 4. You need to choose a toothbrush with a small head and soft bristles to brush your natural teeth. | True |
| 5. Interdental brushes are used to remove the dental plaque that forms between teeth. | True |
| 6. There is no need to consider the size of the interdental brush. | False |
| 7. Fluoride toothpaste for adults with a fluoride content of 1350 ppm or higher can prevent tooth decay. | True |
| 8. The Bass brushing technique involves positioning the toothbrush vertically on the teeth. | False |
| 9. Using moisturizing mouthwash can mitigate dry mouth. | True |
| 10. Older adults require regular dental checkups every 6 months. | True |

Table S2. Items and positive answers of attitudes towards geriatric oral health scale

| Item | Positive answer |
| --- | --- |
| 1. Physical health is more important than oral health. | Disagree |
| 2. Assisting older adults in cleaning their teeth is annoying. | Disagree |
| 3. Oral cleansing is a part of daily body cleaning. | Agree |
| 4. Oral care is as important as physical care for older adults. | Agree |
| 5. I don’t have enough time to assist older adults in cleaning their teeth. | Disagree |
| 6. It is important to take time to assist older adults in cleaning their teeth. | Agree |
| 7. Older adults should be reminded that regular dental checkup is important. | Agree |

Table S3. Items and positive answers of self-efficacy of geriatric oral health scale

| Item | Positive answer |
| --- | --- |
| 1. I am confident about assisting older adults in cleaning their mouths. | Agree |
| 2. I am confident about reminding older adults to clean their mouths after each meal. | Agree |
| 3. I am confident about reminding older adults to clean their mouths before bed. | Agree |
| 4. I am confident about administering appropriate oral care depending on the oral health of the individual older adults. | Agree |
| 5. I am confident about checking the suitability of oral care instruments for older adults. | Agree |
| 6. I am confident about assisting older adults in cleaning their dentures. | Agree |
| 7. I am confident about assisting older adults in using the interdental brush. | Agree |
| 8. I am confident in assisting older adults brush their teeth with the Bass brushing technique. | Agree |
| 9. I am confident in assisting older adults in using the moisturizing rinses. | Agree |
| 10. I am confident about reminding older adults to have regular dental visits. | Agree |

Table S4. Items and positive answers of intention to assist oral care for older adult scale

| Item | Positive answer |
| --- | --- |
| 1. I will take the initiative to remind older adults to perform oral care after each meal. | Agree |
| 2. I will take the initiative to remind older adults to clean their mouths before bedtime. | Agree |
| 3. I will take the initiative to assist older adults in cleaning their dentures. | Agree |
| 4. I will take the initiative to assist older adults in using the interdental brush. | Agree |
| 5. I will take the initiative to assist older adults in using the Bass brushing technique to brush their teeth. | Agree |
| 6. I will take the initiative to recommend proper tools for oral care to older adults. | Agree |
| 7. I will take the initiative to recommend proper tools for oral care to caregivers of older adults (such as family members). | Agree |
| 8. I will take the initiative to check the suitability of oral care tools for older adults. | Agree |
| 9. I will take the initiative to recommend moisturizing rinses to older adults with dry mouth. | Agree |
| 10. I will take the initiative to remind older adults to have regular dental visits every 6 months. | Agree |
