# Supplementary material for: Virtual reality-based simulation learning on geriatric oral health care for nursing students: a pilot study
Source: BMC Oral Health. 2024 May 28;24:627. doi: 10.1186/s12903-024-04249-y (PMC11134768; doi:10.1186/s12903-024-04249-y)
Supplement: Supplementary file 2 — Supplementary Material 2 [file 12903_2024_4249_MOESM2_ESM.doc]

Appendix 2. The flow diagram of study participants

**Allocation**

**Analysis**

**Follow-Up**

**Enrollment**

Assessed for eligibility (n=52)

Excluded (n=2)

#Not meeting inclusion criteria (n=1)

#Declined to participate (n=1)

Analysed (n=25)
#Excluded from analysis (n=0)

Lost to follow-up (n=0)

Allocated to VR intervention (n=25)

Lost to follow-up (n=0)

Allocated to control (n=25)

Analysed (n=25)
#Excluded from analysis (n=0)

Randomized (n=50)

Pre-test (T0)

Week 2 Post-test (T1)

Week 4 Post-test (T2)
