# Supplementary material for: Virtual reality-based simulation learning on geriatric oral health care for nursing students: a pilot study
Source: BMC Oral Health. 2024 May 28;24:627. doi: 10.1186/s12903-024-04249-y (PMC11134768; doi:10.1186/s12903-024-04249-y)
Supplement: Supplementary file 3 — Supplementary Material 3 [file 12903_2024_4249_MOESM3_ESM.docx]

Appendix 3 Descriptive statistics for the questionnaire scores and sample distribution according to floor and ceiling effects (N = 50)

|  | **Mean (SD)** | **Percentiles** | | | **Range** | **Floor effect** | | **Ceiling effect** | |
| --- | --- | --- | --- | --- | --- | --- | --- | --- | --- |
|  |  | **25** | **50** | **75** |  | **n** | **%** | **n** | **%** |
| Knowledge (0; 10) | 6.50 (1.71) | 6 | 7 | 8 | 2; 10 | 1 | 2.0 | 1 | 2.0 |
| Attitude (7; 35) | 28.16 (2.63) | 26 | 28 | 30 | 24; 34 | 5 | 10.0 | 1 | 2.0 |
| Self-efficacy (10; 50) | 34.68 (5.52) | 32 | 36 | 38 | 13; 45 | 1 | 2.0 | 2 | 4.0 |
| Behavioral Intention (10; 50) | 39.40 (5.19) | 36 | 40 | 41 | 29; 49 | 1 | 2.0 | 3 | 6.0 |

*SD* standard deviation
